# Pivot Teeth

**Published:** 1860-10

**Authors:** 


*Pivot Teeth,* when skillfully done, prove to be the most satisfactory of all artificial teeth worn during a period ranging from eight to fifteen years. The fang unnoccupied by the pivot should be filled with gold, the wooden pivot should accurately fit the aperture made for it in the fang, and the artificial crown accurately fitted upon the face of the fang. B.

